# Unusual Cause of Orocutaneous Fistula in the Neck

**DOI:** 10.1155/2012/658536

**Published:** 2012-06-28

**Authors:** Sudipta Saha, Ashesh Jha, Navneet Kaur

**Affiliations:** ^1^Department of Surgery, Lady Hardinge Medical College, New Delhi 110001, India; ^2^Department of Surgery, University College of Medical Sciences and GTB Hospital, Delhi 110095, India

## Abstract

A case of orocutaneous fistula secondary to submandibular sialolithiasis, which was masquerading clinically as branchial fistula is presented. This case highlights the importance of conducting fistulogram in the evaluation of discharging lesions in the neck.

## 1. Introduction

Orocutaneous fistula is a pathologic communication between the oral cavity and cutaneous surface. Orocutaneous fistula can occur because of dental infections, salivary gland lesions, neoplasms, or branchial fistula [1–3]. Sinuses in the neck secondary to the tubercular lymphadenitis also present as discharging lesions [4].

An unusual case of discharging lesion in the neck is presented where the diagnosis was not suspected based on the clinical symptoms. 

## 2. Case

**Figure 1:**
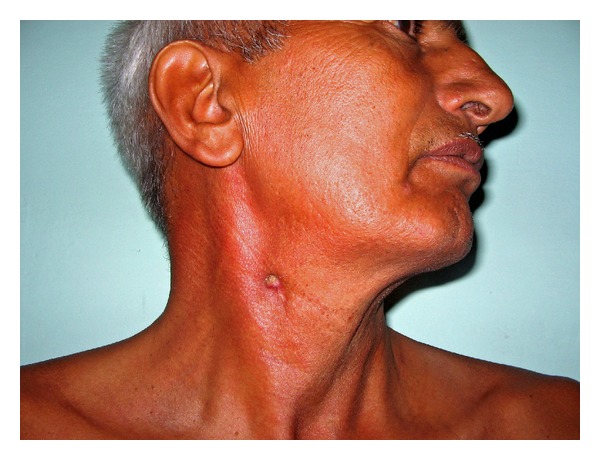
Fistula openining in neck.

**Figure 2:**
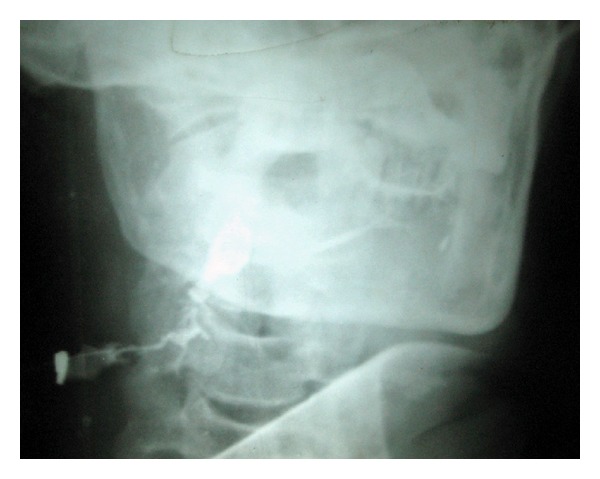
Fistulogram showing the communication of the fistula opening with the submandibular duct (Wharton's duct).

A 54-year-old man presented with small seropurulent discharging lesion located at the junction of upper one-third and lower two-thirds of the anterior border of the sternocleidomastoid muscle (Figure 1). The lesion started as a small nodule three months back which busted after two weeks and resulted in a small ulcer with seropurulent discharge. Patient did not have previous history of swelling in the neck. Examination of the oral cavity was normal. Clinically, the possibility of brachial fistula or tubercular sinus was considered. Biopsy was taken from the fistula, which revealed chronic inflammation and no evidence of tuberculosis. A fistulogram showed that the fistulous tract was communicating with the submandibular duct (Wharton's duct) (Figure 2). No stone was detected on the X-ray. 

The diagnosis of orocutaneous fistula with communication with the submandibular duct was hence made. Excision of the submandibular gland and the fistulous tract was carried out. The tract was found to be communicating with the submandibular duct and a small stone was present in the duct just distal to the point of communication between the fistulous tract and the submandibular duct. Postoperative recovery of the patient was uneventful. 

## 3. Discussion

Salivary gland fistulas are rare. More commonly it results from trauma or iatrogenic injury to the salivary gland or its duct. There are very few case reports of salivary gland sialolithiasis resulting in orocutaneous fistula [5, 6]. 

However, in this particular case patient did not have any symptoms of sialedenitis. Tuberculosis being very common in India, possibility of cervical sinus secondary to the tubercular lymphadenitis was considered but the biopsy from the fistula opening ruled-out tuberculosis. Branchial fistula was the other possibility thought, as they also present as intermittently discharging sinus in the middle or the lower part of the neck [7]. To completely delineate the tract fistulogram was performed, which surprisingly revealed the communication of the fistula with the submandibular duct, with the contrast going into the floor of the mouth at the opening of the submandibular duct. 

## 4. Conclusion

Salivary fistula can rarely present as discharging lesions in the neck and fistulogram should be performed to completely delineate the tract in the cases of neck fistulas.
